# Assessing the health workforce in Afghanistan: a situational analysis into the country’s capacity for Universal health coverage

**DOI:** 10.1186/s13031-025-00663-3

**Published:** 2025-04-17

**Authors:** Narges Neyazi, Nima Yaghmaei, Mirwais Ahmadzai, Elisabeth Kleipool, Nadine Naumann, Myrte Wassenaar, Muhammad Haider Omar, Fethiye Gülin Gedik, Sandra Alba, Marjolein Dieleman, Abdul Ghani Ibrahimi, Alaa AbouZeid

**Affiliations:** 1 World Health Organization Country Office, Kabul, Afghanistan; 2 KIT Royal Tropical Institute, Amsterdam, Netherlands; 3 Kabul University, Kabul, Afghanistan; 4 Ministry of Public Health, Kabul, Afghanistan; 5 WHO Regional Office for Eastern Mediterranean, Cairo, Egypt; 6 Faculty of Medicine, Cairo University, Cairo, Egypt

**Keywords:** Universal health coverage, Afghanistan, Primary health care, Human resources for health, Conflict, Gender, Humanitarian health

## Abstract

**Background:**

Universal health coverage (UHC) is a key component of Afghanistan’s health plan, but the country faces challenges due to decades of conflict and instability. Concurrently, healthcare successes have been achieved despite significant shortages in the health workforce. A fit-for-purpose health workforce is crucial for achieving UHC, and requires decision-making by policy-makers driven by sufficient evidence. This study presents a comprehensive situational analysis of Afghanistan’s health workforce in 2023, focusing on distribution by geography, gender, facility type, as well as trends in health worker production.

**Methods:**

A multi-stage assessment of the active health workforce was conducted through a national census-style count using active registries and facility-level verification through sampled facilities visits. Health worker production was estimated through an analysis of enrollment and graduation figures from public and private institutions from 2019 to 2023.

**Findings:**

We estimated 63,632 health workers in Afghanistan in 2023, with 73% in the public sector and 27% in the private sector. Key health workers (physicians, nurses and midwives) total 10.3 per 10,000 population, falling significantly short of the aspirational UHC threshold (44.5 key health workers per 10,000). Substantial geographic disparity exists between provinces, with remote provinces reporting far fewer key health workers compared to the national average and Kabul representing approximately 50% of the country’s specialized physicians. Significant gender imbalances exist as only 18% of specialized physicians and 29% of nurses are female. Health workforce production is largely dependent on the private sector, and has declined for certain cadres due to restrictions on female education, which are increasing in severity. Majority female cadres, such as Obstetrics/Gynecology, are anticipating significant declines in active staff, jeopardizing aspirations of UHC.

**Interpretation:**

Afghanistan faces critical health workforce challenges, including shortages, gender imbalances and unequal geographic distribution. These findings provide essential insights for policymakers to guide human resources policies aimed at achieving UHC.

## Introduction

### Universal health coverage in Afghanistan

Universal health coverage (UHC) has become a major goal of health reforms in many countries and is a priority objective of the World Health Organization (WHO) [1]. UHC, which is defined by the WHO as “access to key promotive, prevention, curative, and rehabilitative health interventions for all at an affordable cost”, is a key component of the Afghan Health plan [2]. However, the Afghan health system faces significant challenges due to decades of political instability and conflict, the COVID-19 pandemic, and a multitude of other setbacks [3, 4].

A fit-for-purpose health workforce, especially at primary level, is the first step to achieve UHC [5]. This requires informed decision-making by policy makers driven by sufficient evidence on the required human resources for health (HRH) to achieve effective coverage. In particular, information on key health workers (i.e., physicians, nurses and midwives) at primary care level is essential to enable this decision-making.

### The current service delivery system

During the past two decades, the Afghan public health system has been built around the Basic Package of Health Services (BPHS), which focuses on primary health services, and the Essential Package of Hospital Services (EPHS), which focuses on secondary and tertiary care in district and regional hospitals [6]. Specialized tertiary hospitals which provide specialized medical services are not covered by the EPHS however. Currently, health service delivery is provided primarily by contracted non-governmental organizations (NGOs) with stewardship by the Ministry of Public Health (MoPH) [6]. Concurrently, the MoPH regulates numerous private for-profit actors which comprise a sizeable proportion of Afghanistan’s health service delivery and the majority of health expenditure [3, 4].

The BPHS and EPHS programs have played a critical role in the advances in the Afghan public health system since 2001 [7]. However, these achievements have been made despite significant shortages of health workers across these programs [8, 9]. The production of health workers is conducted by both the public and private health education sector coordinated by the MoPH, which oversees health institutes, and the Ministry of Higher Education (MoHE), which oversees universities [10].

### The need for a health workforce assessment

Due to weakness of the Human Resources Management Information System (HRMIS), which was established in 2018 but is yet to scale-up and lacks complete data, there is a dearth of quality data on HRH across the country, particularly in the private for-profit sector [10]. The latest assessment, conducted prior to the government transition using data from 2017 to 2018, suggested three major challenges for HRH in the Afghan health system: a significant shortage of health workers, an overall gender imbalance in the health workforce driven in part by lower numbers of female health worker graduates, and an unequal geographic distribution of health workers [10, 11]. The assessment estimated a national density of 8.75 key health workers (physicians, nurses and midwives) per 10,000 population, thus, indicating a significant shortage compared to the aspirational UHC threshold of 44.5 per 10,000 population [10, 11]. However, these estimates may be outdated due to turbulent events, including conflict, the government transition and the COVID-19 pandemic, which resulted in changes to health worker migration, retention, and attrition [8, 12]. In addition, since the government transition there have been major gender-based education policy changes and labour-restrictions, potentially further impacting the gender imbalance of health workers. Therefore insights into the future output of human resource production is critical [8].

Thus, we present a situational analysis of the health workforce in Afghanistan in 2023, focusing on the distribution of health workers by geography and gender, as well as their distribution between public and private health facilities. We also present trends in the production of these health workers, particularly key health workers (physicians, nurses and midwives). The aim of this analysis is to complement the existing HRMIS and present decision makers with the necessary information to guide HRH policy to achieve UHC.

## Methodology

The present investigation consisted of two components based on the health labor market (HLM) framework (production of health workers through education and the existing pool of health workers) [13].

### Active health workforce assessment

In the first component, health worker estimates were obtained through a multi-stage process. The first stage was a national level census-style count of the active workforce, defined as having been on payroll records in the past month. Where these records did not exist, such as in the private health sector, active registry data was utilized, although there was often poor record keeping. Public service providers (such as NGOs) and the MoPH private sector coordination directorate provided national data on the number, gender, and cadre of active health workers.

In the second stage, a sample of health facilities were visited to estimate a verification coefficient to adjust the information collected in the first stage from the respective facilities. This was considered necessary to address any inaccuracies due to outdated information in payroll or staff databases. The verification coefficient was calculated as the ratio of staff counts in the visitation phase for each major cadre (i.e., non-specialized physicians, specialized physicians, nurses, midwives and others) versus staff counts in databases. 3,282 individual public facilities and 861 private facilities were included in the sample frame. Many of these facilities have multiple individual service providers (such as multiple NGOs) present in the same facility. Twelve provinces were selected using stratified sampling based on the level of health service accessibility for the population provided by the MoPH, and six districts were selected per province using PPS (Probability-Proportional-to-Size) sampling based on the number of facilities in the district. Public facilities were then randomly sampled using a simple sampling frame. Private facilities present in the sampled districts were visited for verification if location information was available at the Provincial Public Health Directorate.

The sample frame for public health facilities was the most reliable as the implementation of health services provided by these facilities is overseen by the government and NGO network. This allowed for a complete follow-up of sampled facilities. Although clinical private facilities are required to report to the Provincial Public Health Directorate, the registry can contain out of date information on the status or location of the health facility. In the sampling process, all listed private facilities were assessed and information on the location and governance of the private facility was collected in order to facilitate the verification process. The registry of non-clinical private facilities, such as diagnostic clinics, was of poor quality, and often data from these facilities was aggregated to provincial level in the national records, preventing them from inclusion into the sampling frame, and therefore excluding them from the verification process as well. Thus, the active choice was made to exclude non-clinical private facilities from the verification process, therefore preventing these facilities from reporting with confidence intervals. Required sample sizes (356 public health facilities and 334 private health facilities) were calculated to estimate a single proportion of the facility verification coefficient with the following assumptions: Z-score (1.96), margin of error (0.05), and an estimated proportion of the verification coefficient (0.5). Overall, the sample was powered for analysis at the national level for both private and public facilities independently, while disaggregation by facility type and province was done for exploratory purposes.

Furthermore, dual practice (i.e., when a health worker is simultaneously active at both public and private facilities) was assessed through a set of manager interviews at private facilities [14]. This allowed for the estimated quantification of dual practice amongst health workers between the public and private sector to adjust total health workforce estimates and avoid double counts. To account for potential differences in the reported rate of dual practice amongst health workers across cadres, reporting for dual practices was done for each cadre type, allowing for cadre specific adjustments in the analysis phase. Additionally, to account for information bias driven by social desirability from health managers, the anonymity of the health facility to the facility sample and staff members to the overall sample was expressed in the lead up to the section in the questionnaire.

### Health workforce production assessment

To obtain an estimate of the health workforce production, an assessment was conducted using the databases of the MoHE and MoPH for both public and private training institutes and universities. The administration of MoHE and MoPH educational institutes are conducted separately and therefore the databases were accessed separately. Enrollment and graduation figures of students disaggregated by faculty, specialization, and gender were collected retrospectively from 2019 to 2023.

### Data management and analysis

All data was collected between June and September of 2023. All data on health workforce counts was collected using digital devices with pre-programmed questionnaires using Open Data Kit (ODK) [15]. Health education data was collected via Excel, while private education institutes visits were conducted using ODK. All data was stored on a centralized GDPR-compliant server to which only the data manager and technical manager had full access.

Data analysis was conducted using Stata, Excel, and maps were produced with QGIS. The national estimates of health workforce were calculated by aggregation of all national data, followed by disaggregation by facility type, province, and gender. Point estimates for the verification coefficient included confidence intervals and were adjusted for by sample weights. These point estimates and confidence intervals were then applied to the respective health staff cadres for the final counts. Due to insufficient location information in the sampling frame of the private facilities, there was a smaller final sample size for private facilities in the verification phase (55 facilities). Therefore to adjust for potential sampling bias and to quantify uncertainty, we applied Bootstrapping technique [16]. This technique performs a large number of simulated samples by resampling the original dataset thus accounting for the variability and producing robust confidence intervals for the private facility sample, which were underrepresented. Furthermore, all the national estimates were corrected for the proportion of dual practitioners at both private and public facilities by utilizing the calculated estimates of dual practice and reducing health worker counts by estimated dual practice for the specific cohort. Annual health worker production estimates from the education component were visually analyzed using time series plots to identify trends.

### Ethics

This study did not collect personal or sensitive information. WHO country office obtained the IRB approval (IRB Code No: A.0823.417) from Afghanistan National Public Health Institute (ANPHI). Health manager interviews were the only form of primary data collection. Informed verbal consent was obtained from all health managers prior to participation in any surveys. All data collected was anonymous, all participation was voluntary, and no questions related to personal experiences.

## Results

### Active health workforce

Table 1 presented the estimated workforce counts by cadre, along with confidence intervals derived using the verification coefficients. Estimated counts are presented by cadre and for the public and private sector. There are an estimated 63,632 health workers in Afghanistan in 2023 of which 46,397 (73%) are public health workers and 17,236 (27%) are private health workers (Table 1). Of the estimated total public health workers, 28,583 (62%) are employed in BPHS facilities and 17,815 (38%) are in EPHS facilities. There are approximately three times as many specialized physicians in EPHS facilities compared to BPHS, while there are more than twice as many midwives in BHPS facilities compared to EPHS.

Overall, key health workers (physicians, nurses and midwives) account for 55% of all health workers. Amongst key health workers, nurses in public and private facilities account for approximately 39%, while physicians accounted for 38%, and midwives accounted for 24%. The public sector accounted for 83% of midwives (6,746), 78% of nurses (10,441), and 67% of physicians (8,839) (specialized and non-specialized). However, among the physicians, the specialists are more likely to be private sector staff (45%) than non-specialized physicians (25%). Overall, the confidence intervals presented in Table 1 suggest the public sector estimates are more precise than the private sector due to the larger achieved sampled sizes in the verification stage of data collection and the more consistent health worker counts in the public sector.

**Table 1:** Active health workforce estimated counts and confidence intervals presented by cadre for the public and private sector

	Public						Private			
	BPHS			EPHS			Hospitals/Clinics			Other
Health Workforce Counts	#	CI Lower	CI Upper	#	CI Lower	CI Upper	#	CI Lower	CI Upper	#
Physician (Non-Specialized)	3110	2739	3481	2935	2585	3285	1931	1555	2308	123
Physician (Specialized)	715	629	800	2079	1831	2327	2254	1815	2694	8
Nurse	5066	4642	5490	5375	4925	5825	2831	2119	3543	57
Midwives	4638	4284	4991	2108	1947	2269	1337	1004	1669	11
Allied Health Worker	15,054	13,318	16,790	5318	4704	5931	3415	2727	4103	5268
	Public						Private			
	BPHS			EPHS			Hospitals/Clinics			Other
Specialist Physician Counts	#	CI Lower	CI Upper	#	CI Lower	CI Upper	#	CI Lower	CI Upper	#
General Surgeon	132	116	147	384	338	430	382	307	456	1
Internal Medicine Specialist	71	63	80	255	224	285	418	673	999	2
Pediatrician	49	43	54	254	223	284	315	253	376	1
OBS/GYN	39	34	43	239	210	267	373	300	446	2
All Other Specialists	419	369	469	947	834	1060	766	617	916	11

The largest group of health workers in the public sector are allied health workers, of which 35% are vaccinators (7,236) and 3,519 are nutrition counselors (17%), both of which are almost exclusively public sector health workers. In the private sector the largest cadres are pharmacy technicians (3,187) and laboratory technicians/technologists (2,958) who are primarily employed in non-clinical private facilities.

A total of 5,056 specialized physicians were reported in the country, the largest group of these specialists are general surgeons (899), followed by internal medicine specialists (746) and obstetricians/gynecologists (OBS/GYN) (652). The remaining 42% of specialists are distributed through the other 28 categories of specialists available in the country. Both private facilities and EPHS facilities employ significantly more specialists than BPHS facilities across all types of specializations. Although this disparity is present in specializations that relate to services provided in secondary and tertiary facilities, such as internal medicine specialists, the highest level of disparity observed is for obstetrics/gynecology (OBS/GYN), as for every OBS/GYN specialist in BPHS facilities (39) there are approximately 6 in EPHS facilities (239) and 10 in private health facilities (373).

#### Gender distribution

**Fig. 1:**
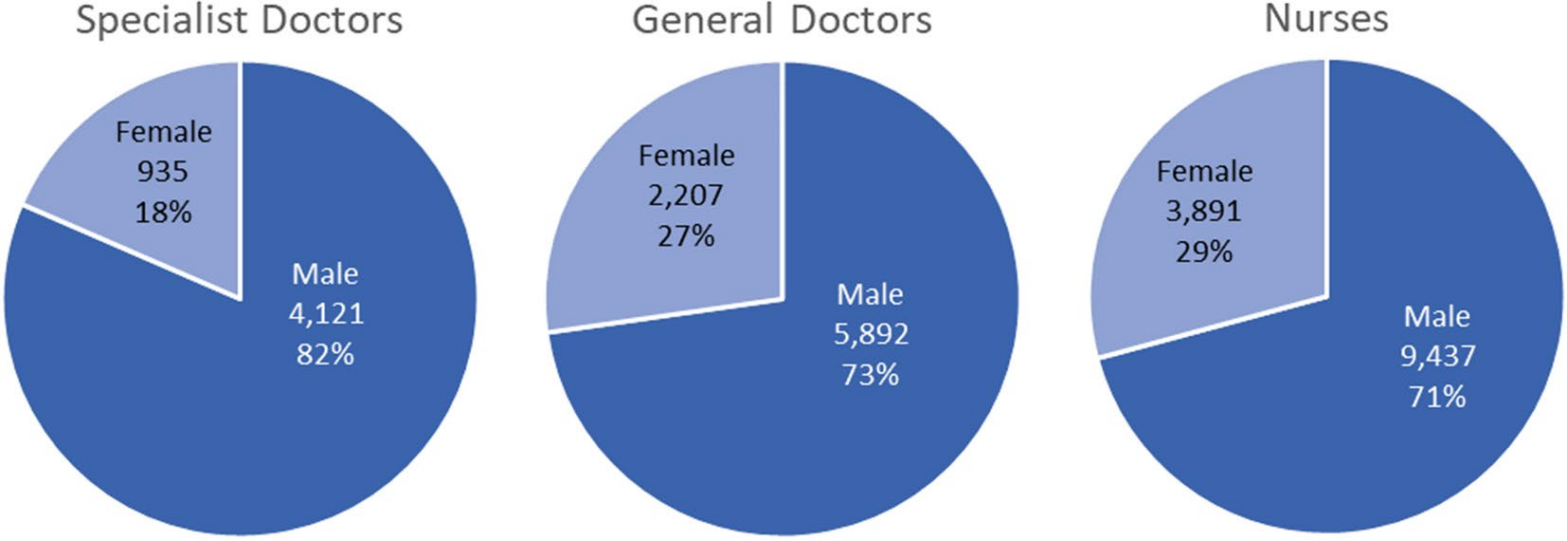
Gender distribution of health cadres

Health worker estimates indicate an unequal gender distribution depending on the cadre and specialization. Figure 1 demonstrates that only an estimated 27% of non-specialized physicians and 18% of specialized physicians are female. Furthermore, 29% of all nurses are female. Of all female specialist physicians, 69% are OBS/GYN. Overall, males account for the majority of health workers, except for midwives who are all female.

#### Distribution of health staff by Province

**Fig. 2:**
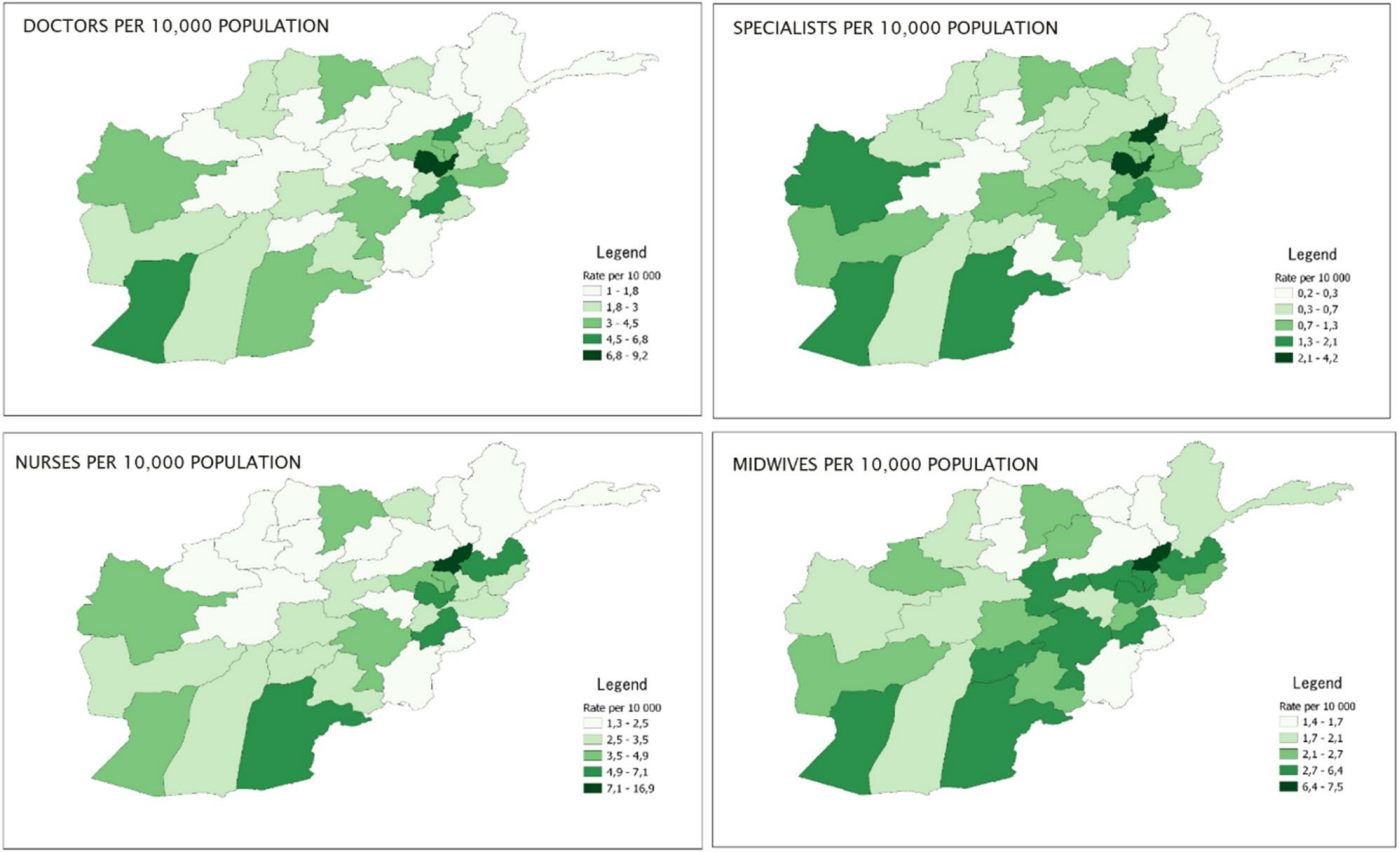
Density of key health workers per 10,000 population by province

There is an uneven distribution of health workers across the country. For each of the key health worker types (physicians, nurses and midwives), Kabul province has the highest absolute number of staff, with 5,313 non-specialized physicians (41% of the national count), 2,427 specialized physicians (48%), 4,120 nurses (31%), and 1,734 midwives (21%). This is despite Kabul accounting for 17% of national population [17]. Other high population provinces, such as Herat and Kandahar, also have relatively high absolute counts. Figure 2 presents key health workers (physicians, nurses and midwives) densities per 10,000 population. There is high heterogeneity in the availability of key health staff across the provinces. Only Kabul and Panjsher consistently reported higher ratios of key health staff availability, while some of the southern provinces and provinces close to Kabul have ratios higher than the national average. More remote provinces in the center and northern regions, reported the lowest ratios. For example, Badakhshan reported only 1 physician per 10,000 population, while Faryab reported 1.3 nurses per 10,000 population. Overall, specialist physicians had the most unequal geographic distribution amongst key health workers, while nurses and midwives had the most equal distributions.

**Fig. 3:**
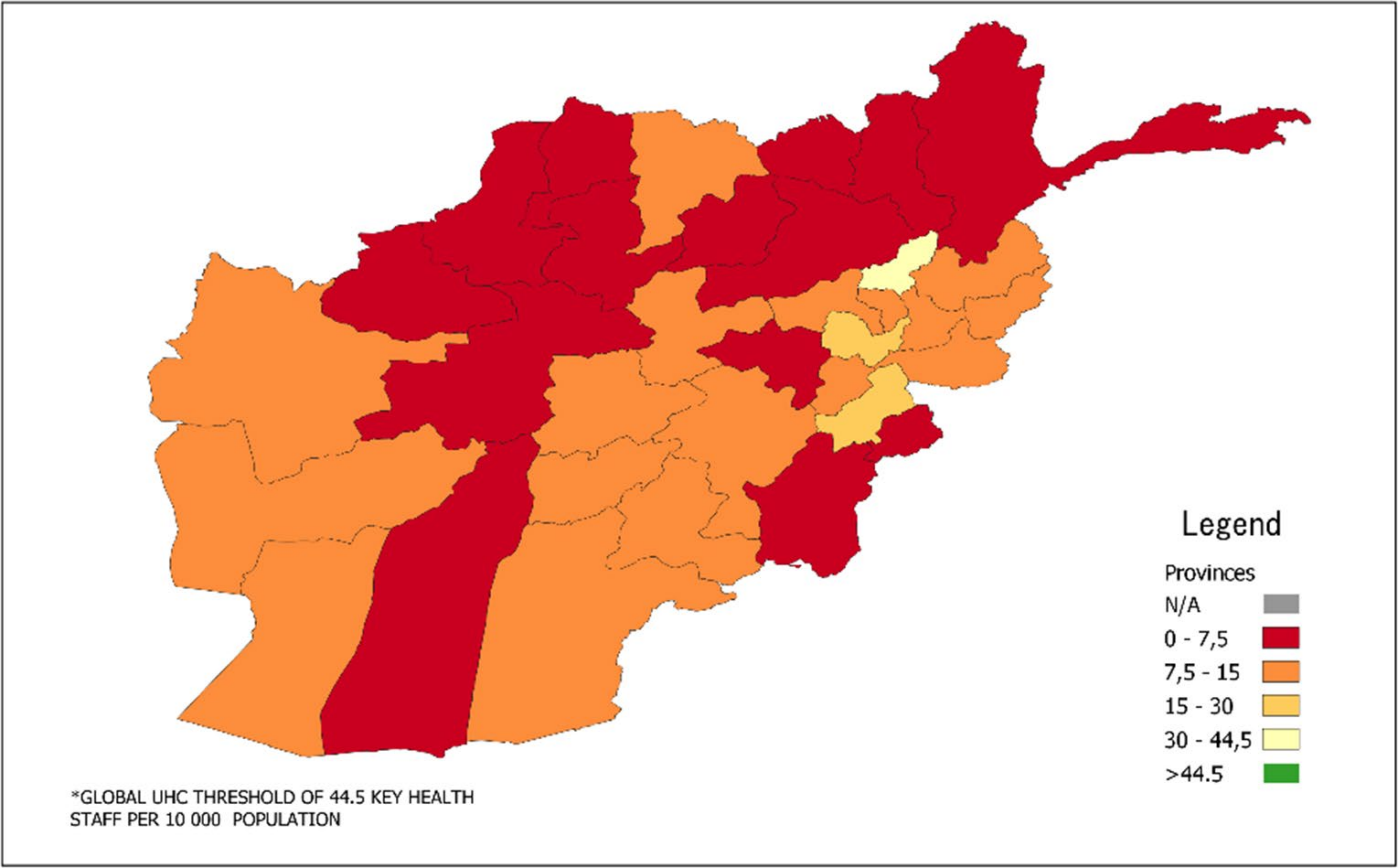
Distribution of Total Key Health Staff (Physicians, Nurses and Midwives) by Province Compared to aspirational UHC Threshold (Ratio of Key Health Staff per 10,000 Population)

Compared to the aspirational UHC thresholds of 44.5 key health workers (physicians, nurses and midwives) per 10,000 population, additional key health workers are required for every province, (Fig. 3). Panjsher province has the highest density of key health workers (32.7 per 10,000 population), followed by Kabul (19.4 per 10,000). Although these figures fall short of the aspirational UHC threshold, they are significantly higher than the national level of 10.3 per 10,000. Overall, 14 provinces have less than 7.5 key health workers per 10,000, which is considered alarming compared to the national level. In total, approximately 115,000 additional key health workers are required in Afghanistan to approach key health workers figures required for UHC.

### Health workforce production assessment

All female enrollment in university-based programs, as well as medicine studies in both the public and private sector institutes, were banned in 2023. Only health programs based in institutes mandated by the MoPH, such as midwifery and nursing, were open for female enrollment. Therefore, this study found that there are no females enrolled in medicine or university-based health programs. Overall, health workforce production is largely dependent on private education facilities, as the private sector accounts for 98%, 94%, and 80% of midwifery, nursing and medicine enrollments respectively. Enrollment figures are presented in the results as they have the most recent data, however, final production figures are dependent on the number of graduates. On average, approximately 50% of students enrolled in medicine, nursing, and midwifery graduate from their program.

**Fig. 4:**
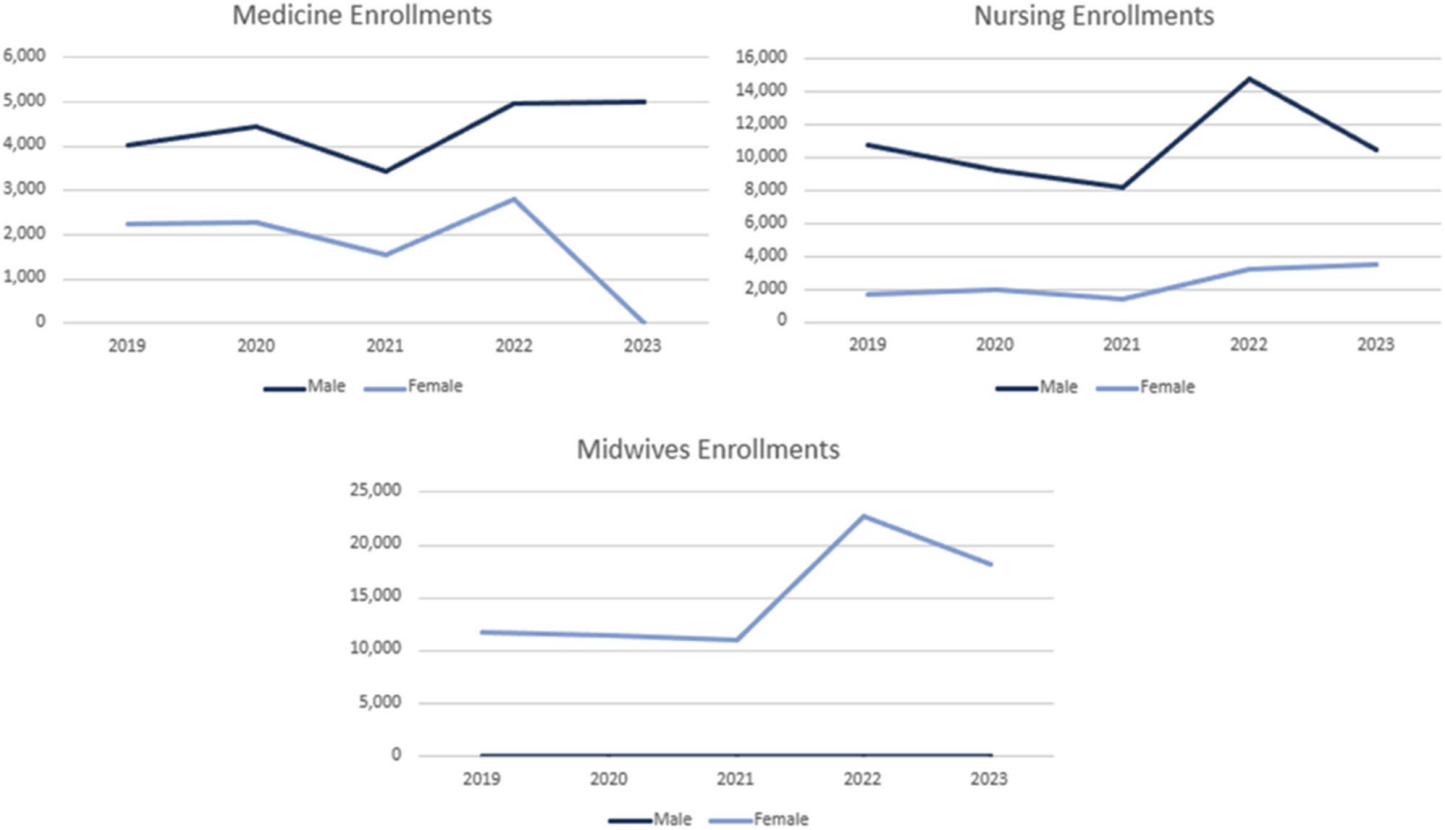
Education enrollments (Public and Private)

#### Medicine

Enrollment in medicine programs (Fig. 4a) demonstrated relative consistency for both male and female students between 2019 and 2022, with an average of 4,226 male enrollments and 2,221 female enrollments per year. In 2023, male enrollments rose to 5,323, however, due to the complete ban on female enrollment, the total medicine student enrollment in 2023 (5,323) is lower than the yearly average of the previous four years (6,447). With the current ban on enrollment of females, it is anticipated that female graduations from both public and private education facilities will remain at zero for the foreseeable future. The increase of male enrollments is likely insufficient to replace the expected decline of female graduates.

#### Nursing

In 2022, nursing enrollments (Fig. 4b) peaked at a total 18,117 enrollments compared to the average of 11,118 enrollments in the previous three years. On average, between 2019 and 2022, 84% of nursing enrollments were male. Thus, despite a slight increase in overall female nursing enrollment in 2023, overall nursing enrollment counts reduced to 14,051 in 2023 due to a reduction in male enrollment.

#### Midwifery

At the time of the study, female enrollment was allowed in midwifery programs run by the MoPH. As seen in Fig. 4c, midwifery enrollment increased from 2019, peaking at over 22,000 students in 2022. Since the graduation rate for midwives are 50%, approximately 9,000 midwives are expected to graduate per year if enrollments were to remain consistent with 2023 figures.

## Discussion

### Health workforce shortages

The results presented highlight the critical challenges that remain in Afghanistan’s health workforce in order to achieve UHC. In total, there is an approximately estimated figure of 35,000 key health workers (physicians, nurses and midwives), and thus an additional 115,000 would be required to achieve the aspirational UHC thresholds. In addition to the standard UHC thresholds, there are other health workforce targets which either have been applied to specific contexts or were developed for alternative health service delivery standards (such as minimal threshold for critical service delivery) [18, 19]. In the current context, Afghanistan is far from achieving aspirational threshold regardless, and therefore from a pragmatic perspective this study compared against the standard UHC threshold.

Although the health workforce to population rate differed by cadre across provinces, for this study, we draw attention to the overall shortage, as across all provinces and across all cadres, health workforce shortages were observed. Furthermore, there is a gender imbalance for doctors and nurses, and an unequal geographic distribution of health workers. Certain provinces, particularly Kabul, account for a disproportionate number of health workers in certain cadres, particularly specialist physicians. Finally, there is an insufficient production of health workers from public education facilities, which is further threatened by the ban on female enrollment.

In addition to Kabul province, within other provinces, the urban centers, particularly provincial capitals, account for a large proportion of private health facilities, specialized physicians, and key health workers. Secondary and tertiary facilities, such as those related to the EPHS program, and private facilities are more concentrated in urban centers, therefore key health workers, and in particular specialized physicians, are inequitably distributed throughout the country. These findings correspond with the inequitable distribution of health workers in neighbouring countries and remote regions of geographically diverse settings similar to Afghanistan [20–22]. In the Iranian province of Sistan and Baluchestan researchers identified that nurses and midwifes were somewhat equitably distributed, while physicians, particularly specialists, were inequitable in distribution [20]. Researchers in Indonesia demonstrated despite the unequal distribution of health workers between provinces, intra-provincial distributions were even less equal, suggesting that the largest differences are between urban and rural regions, not between provinces [22].

Therefore, despite the poor health worker ratios in most provinces, these figures may be significantly worse when assessed at a sub-provincial level. It is recommended that further investigations be conducted into the factors associated with the poor distribution of inter and intra-provincial health workers.

### Health workforce production

The production of health workers shows concerning trends. For example, nearly all OBS/GYN specialists are female. However, currently there are no females being trained in medicine or other university-based allied health programs. Thus, it is clear that in a short period of time the number of female physicians and other allied health workers will begin a sharp decline jeopardizing the country’s health system. This regression comes in the context of decades of improvement in the health workforce, particularly in female oriented roles, such as midwives who have become the backbone of many primary health services [23, 24].

Furthermore, the production of nurses and physicians from public institutes, regardless of gender, is not close to sufficient. The private sector produces considerably more health workers, however, there is limited insights into the retention, distribution, and quality of the graduates. To date there is limited evidence on the quality of employed health workers who have received training in private institutions, nor is there evidence on the retention and distribution rates of private institution graduates compared to public institution graduates. However, one study comparing the quality of pre-service midwifery education in private vs. public institutions identified underperformance in private institutions, as public institutions adhered more strictly to the curriculum and provided better hands-on clinical training placements [25]. Furthermore, recent policy changes in regard to gender and education have created further challenges to the operations private institutions, potentially jeopardizing their quality [26].

Overall, the combined graduation figures of private and public health programs may give indication that there is sufficient health workforce production, however the active health workforce counts suggests that this does not translate into a sufficient workforce, likely due to poor retention and high turnover rates, low employment rates and the geographic distribution of graduates [27].

### Policy recommendations

#### Human resources for health strategy

A number of provinces present acute shortages in health workers that requires heightened attention and targeted action. Priority should be placed on producing a pool of quality healthcare workers across cadres. Concurrently, efforts should be made to recruit and retain these individuals within the system, and if possible, encourage the distribution of these individuals to underprivileged provinces. The relocation of health staff to provinces with acute shortages may provide some support, however, these relocations may risk shifting shortages elsewhere, including Kabul which has its own staff shortages despite accounting for such a large proportion of the national health workforce.

In addition to relocation of staff, other workforce optimization measures may be considered. These include optimization strategies with the existing health workforce, such as task-shifting, or strategies to retain or attract workforce, such as workplace safety, competitive salary, providing professional development opportunities, and non-financial incentives [11, 28]. Although the health workforce shortage across all cadres of key health workers is a major barrier to the delivery of quality health services in Afghanistan, evidence from other conflict-affected countries indicates that such workforce optimization strategies ameliorate health workforce shortages and fatigue [28]. In addition, community health workers (CHWs) could alleviate pressure and contribute to improved productivity by collaborating with facility-based health workers for basic task shifting. CHWs are not captured in this study as they are not conventionally trained health workers and therefore not considered part of the qualified active workforce. However, there are programs throughout Afghanistan which involve health service delivery via CHWs, particularly in rural and remote areas [29–31]. The involvement and further training of CHWs would be beneficial at this current juncture as health workforce production is hampered by policies [30]. To achieve success policy makers should consider approaches to improve incentives and reduce attrition [29].

Achieving UHC requires a comprehensive strategy focusing on increasing the numbers of key health workers for primary healthcare (BPHS) since the primary level is where 90% of all essential health services are provided [11]. Despite this outsized role of providing essential services, BPHS facilities had significantly fewer physicians, with a particular shortage of specialists for critical services, such as obstetricians. A specific focus on nurses and midwives may be particularly fruitful as the distribution of nurses and midwives was more equal across the country, providing the opportunity to compensate for the concentrated distributions of physicians. In addition, nurses and midwives are more female-oriented roles, helping balance the gender disparity in health facilities across the country. The reintroduction of female enrollment, particularly for key health cadres and the promotion of further female education can help to halt the anticipated reduction of female key health workers and prevent a loss of all OBS/GYN. These health workforce expansions should be prioritized for underserved provinces and districts, ensuring equitable geographic distributions.

Additionally, minimum health worker thresholds should be tailored to the specific needs of particularly challenged provinces. This pragmatic approach sets more achievable targets, addressing the unique healthcare demands of each region while working within the constraints of available resources. Furthermore, the MoPH should improve organizational structures (such as licensing and accreditation) that ensure the private health sector is well monitored and regulated and responsive to the country’s needs.

Regulatory measures and accreditation processes in the private health education sector should be improved to establish standards, ensuring the quality and credibility of graduates from private institutions have the same competencies and performances as the graduates from public institutions, and enabling seamless collaboration with the public sector.

The MoPH (the governing body in responsible for the health system) and the MoHE (the main governing body responsible for the production and recruitment of health workforce), along with critical international partners and donors already engaged with the MoPH in policy development, such as the WHO, UNFPA, UNICEF, and the World Bank, should utilize the body of evidence presented in this study, and other existing evidence, to develop HRH strategies and policies to deliver the health workforce required in terms of quantity and quality [32]. These strategies should take into account the current pool of workers, production rates, retention rates, emigration rates, and other factors and challenges which affect the pool of Afghan health workers. This study does not address the demand-side of health services, and thus further insights on the demand-side, such as health service utilization rates, demographics, and burden of disease, would further assist tailoring HRH strategies. It is critical that in the current HRH challenged environment that exists in Afghanistan, HRH strategy should be evidence-informed.

#### Human resource management information system

A robust Human Resource Management Information System (HRMIS) with coverage of the active workforce in the private and public sector, and coverage of the national production of health workers would serve as a strong platform for evidence informed decision making by policy makers. Human resource assessments, such as this specific assessment, act as a stop-gap in the absence of a robust HRMIS, providing a cross-sectional snapshot of the national circumstances. However, these assessments suffer from inefficiencies, such as inconsistent methodologies. Therefore, it is recommended to further develop the HRMIS by strengthening the quality and scaling nationally. Assessments, such as the one performed for this study, should be used as an example or starting point for further improvements of the HRMIS, as certain initial steps, such as the mapping of data sources and service providers has already been conducted. The HRMIS should be established in each province with key focal points, and priority should be placed on rural and low resource provinces [10, 33]. Furthermore, particular focus should be placed on the weakest element of the HRMIS: the private sector. Overall, a strengthened and national integrated HRMIS using standardized mechanisms will help overcome many of the evidence gaps that exist for policy makers today.

### Limitations of the study

A number of limitations should be kept in mind when considering the findings of this study. All data from health facilities and education institutes were reliant on the quality of the record keeping. Due to logistical constraints, not all provinces and facilities could be visited in the verification process of the health workforce counts. With this consideration, an aggregated verification coefficient was computed which may not reflect provincial variability. Furthermore, although public health facilities had a full sampling frame, private health facilities did not, resulting in a smaller sample size for private facilities. Non-clinical private facilities did not have a sampling frame of sufficient quality, often being aggregated to provincial level. Therefore, nationally, most non-clinical private facilities had staff counts that were provincially aggregated counts of facilities and health staff. As a result, these facilities could not be independently verified, and thus could not be reported with adjusted coefficients. Consequently, we believe that health staff members from non-clinical private facilities may be over-reported.

Total health worker counts were adjusted for dual practice, a common phenomenon in Afghanistan. Dual practice was quantified during the manager interviews. However, these figures are reliant on reporting from health managers, which may be affected by information bias driven by social desirability or recall bias. It is possible that health managers are incentivized to underreport dual practice or that staff members do not disclose to health managers their personal dual practice activities. Furthermore, CHWs could not be included in this study, since they are not recorded within the same databases as conventionally trained health workers, such as doctors or nurses. In addition, the reporting structure of the CHW programs does not allow for data collection at facility level during visits, nor are CHWs reported in pay slips since they do not receive salary. Therefore, the involvement of CHWs in the health system, and their particular contribution to rural and remote areas, could not be demonstrated in this study.

## Conclusions

Achieving UHC in Afghanistan requires the equitable distribution of key health workers (physicians, nurses and midwives) across the public and private health sector. The evidence indicates that at present there are still three major HRH challenges impeding the Afghan health system’s ability to achieve UHC: there are too few health workers at national and provincial levels, as well, there is an unequal geographic and gender distribution of health workers. Therefore, it is recommended to improve the distribution and increase the number of health workers in the public and private sector, particularly the key health workers.

Furthermore, accreditation and low retention levels of private health education institutes should be investigated and outputs from public education institutions should be increased. It is crucial to address female enrollment restrictions to prevent the loss of critical services. Finally, it is recommended to nationally scale and strengthen the human resource management information system in order to drive evidence-informed policy making. All these efforts should be driven by an HRH strategy spearheaded by the MoPH and key international partners and donors, such as the WHO and World Bank.

## Data Availability

No datasets were generated or analysed during the current study.
